# Emergency Center Curbside Screening During the COVID-19 Pandemic: Retrospective Cohort Study

**DOI:** 10.2196/20040

**Published:** 2020-07-21

**Authors:** Alexandra Halalau, Jeffrey Ditkoff, Jessica Hamilton, Aryana Sharrak, Aimen Vanood, Amr Abbas, James Ziadeh

**Affiliations:** 1 Beaumont Health Royal Oak, MI United States; 2 Oakland University William Beaumont School of Medicine Rochester, MI United States

**Keywords:** COVID-19, emergency center, curbside testing, drive-through testing, pandemic, public health

## Abstract

**Background:**

Coronavirus disease (COVID-19) is a global pandemic that has placed a significant burden on health care systems in the United States. Michigan has been one of the top states affected by COVID-19.

**Objective:**

We describe the emergency center curbside testing procedure implemented at Beaumont Hospital, a large hospital in Royal Oak, MI, and aim to evaluate its safety and efficiency.

**Methods:**

Anticipating a surge in patients requiring testing, Beaumont Health implemented curbside testing, operated by a multidisciplinary team of health care workers, including physicians, advanced practice providers, residents, nurses, technicians, and registration staff. We report on the following outcomes over a period of 26 days (March 12, 2020, to April 6, 2020): time to medical decision, time spent documenting electronic medical records, overall screening time, and emergency center return evaluations.

**Results:**

In total, 2782 patients received curbside services. A nasopharyngeal swab was performed on 1176 patients (41%), out of whom 348 (29.6%) tested positive. The median time for the entire process (from registration to discharge) was 28 minutes (IQR 17-44). The median time to final medical decision was 15 minutes (IQR 8-27). The median time from medical decision to discharge was 9 minutes (IQR 5-16). Only 257 patients (9.2%) returned to the emergency center for an evaluation within 7 or more days, of whom 64 were admitted to the hospital, 11 remained admitted, and 4 expired.

**Conclusions:**

Our curbside testing model encourages the incorporation of this model at other high-volume facilities during an infectious disease pandemic.

## Introduction

The first case of human-to-human transmission of coronavirus disease (COVID-19) in the United States was reported on January 30, 2020 [1]. Soon after, in March 2020, the World Health Organization (WHO) declared COVID-19 a pandemic [2]. As of May 25, 2020, there have been 54,679 cases and 5228 deaths across all counties in Michigan [3]. Beaumont Health, the largest 8-hospital health system in Southeast Michigan, has diagnosed more than 7000 COVID-19 patients.

In light of this, as of March 2020, potentially overwhelming numbers of patients were expected to seek care at emergency centers (EC). In the setting of an infectious disease pandemic, this would have resulted in two major problems: (1) cross-infection and (2) additional stress on already overburdened ECs [4]. Accordingly, Beaumont Health set up one of the first EC curbside screening sites early in March 2020 in response to the COVID-19 pandemic.

There is no data yet about the curbside experience in the United States. We describe in detail the curbside screening process and patient outcomes, including EC visits for evaluation, admissions, and mortality. We hope that this information will help other health systems implement similar processes early, safely, and efficiently.

## Methods

EC curbside services were implemented at all 8 hospitals at Beaumont Health during the COVID-19 pandemic. We report the curbside experience from the largest hospital in the system, Beaumont Hospital, in Royal Oak, MI, from March 12, 2020, to April 6, 2020.

The study protocol was approved by the Institutional Review Board at Beaumont Health System.

### Preparation Phase: Project Planning

Beaumont Health anticipated a surge of patients, so the implementation of a screening process became a priority for the health system. We obtained the appropriate approvals within 24 hours and created a multidisciplinary team of health care workers predominantly from the EC, including physicians, advanced practice providers (APPs), residents, nurses, technicians, and registration staff. Additional redeployment of APPs from the inpatient setting helped supplement staffing as needed. An organizational structure for traffic control and security was developed. We chose the EC location as we knew that many patients would be driving up to the EC to seek care. Patients were registered as active EC patients, and documentation was done via the electronic medical record (EMR), including a provider note. All aspects of this process were compliant with the Emergency Medical Treatment and Labor Act and adhered to the Centers for Medicare & Medicaid Services guidelines [5] regarding medical screening exams conducted in an alternate site of care. Data were automatically extracted from the EMR.

### Implementation Phase

#### Pilot Phase

Beaumont Health began curbside testing on March 12, 2020, at its largest campus in Royal Oak, MI. The service was then expanded to the other hospital-based ECs in the health system. Testing was done with real-time reverse transcriptase–polymerase chain reaction (RT-PCR) assay of nasopharyngeal swab. A website was developed to better inform patients about curbside testing and its process and to display patient wait times at each location [6].

#### Curbside Experience at Beaumont Hospital

**Figure 1:**
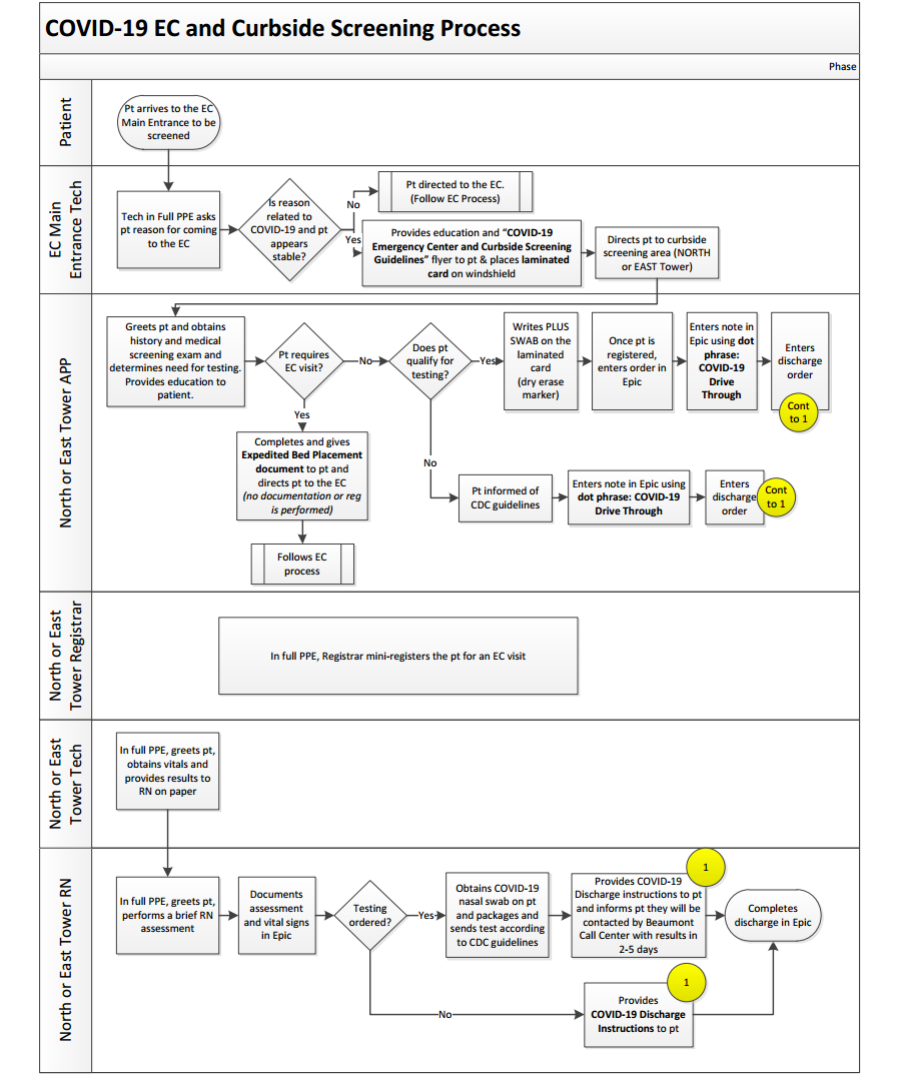
Layout of the emergency center curbside screening process at Beaumont Hospital in Royal Oak, MI. EC: emergency center; CDC: Centers for Disease Control and Prevention; PPE: personal protective equipment; COVID-19: coronavirus disease; APP: advanced practice provider; RN: registered nurse.

Patients required no referral and remained in their vehicle during the entire curbside screening process. In summary, patient flow (Figure 1) started with the EC main entrance tech personnel, who directed patients to a designated curbside location (East or North Tower). The patient would then see an APP, registrar staff, tech staff, and finally a registered nurse, who would eventually discharge the patient. All patients seeking emergency care were initially asked by the EC tech personnel if testing was the purpose of their visit, and if so, they were sent to the curbside location. After a few days, the EC tech personnel was replaced by a midlevel provider or a resident who stayed at the front door and triaged patients to either curbside screening or EC admission. A laminated card was placed on the windshield. APPs carried a dry erasable marker and marked initials on the laminated sheet to indicate who was caring for which patient as registration was occurring simultaneously.

Testing was done based on system capabilities. Initially tests were readily available. Later on, as testing capacity became scarce, we were only able to perform screening for a higher level of care, which meant, based on the Michigan Department of Health instructions [3], that testing was offered if patients experienced moderate cough or fever over 100.4°F, and if the patients had chronic kidney disease; heart disease; diabetes; chronic lung disease; were receiving immunosuppression medication, or were immunocompromised due to cancer treatment, recent surgeries, or other conditions, suggesting high risk for severe disease. As volumes grew rapidly, we moved the location to a main hospital entrance that was not being used during the COVID-19 outbreak, which allowed a reprieve from the weather and increased operational capacity (hot, warm, cold zones; electrical access, etc). We were able to see a large volume of patients without backing up the main emergency department entrance. Also, to avoid long wait times, we opened multiple triage and screening locations based on the surge of patients and also streamlined documentation, increased staffing, and processed in parallel instead of serially.

All patients were discharged home with instructions pertaining to COVID-19. Initially physicians called patients to provide test results, but later the process was transferred to a central location within the health system. During peak volume, there was a 7-hour wait to reach the front of the curbside line until further improvements were made to the process.

#### Personnel Duties, Personal Protective Equipment, and Hygiene Rules

APPs did not come into contact with patients but screened them from outside the car for history and general appearance and reviewed vital signs. Gloves were removed and hand hygiene performed before entering the warm zone for documentation; hand hygiene and new gloves were used before returning to the outdoor area. Personal protective equipment (PPE) comprised the following: N95 mask, face shield, surgical mask, gown, and gloves. Nursing personnel only came into contact with patients if performing nasopharyngeal swab. Proper doffing after swab was obtained and all PPE was changed except for the N95 mask. Of note, at peak volume times, we had 1-2 nurses dedicated to doing swabs. PPE comprised the following: N95 mask, face shield, surgical mask, gown, and gloves. Tech staff performed vitals. Hand hygiene and changing of gloves were performed in between patients. PPE comprised the following: N95 mask, surgical mask, gown, and gloves. Registration staff changed gloves and performed hand hygiene in between patients. PPE comprised the following: N95 mask, surgical mask, gown and gloves.

## Results

### Process Analysis

A total of 2782 patients were seen through the EC curbside at the Royal Oak campus during a period of 26 days. A nasopharyngeal swab was performed on 1176 patients (41%), which came back positive for 348 patients (29.6%). The median time for the entire process (from registration to discharge from the electronic medical system) was 28 minutes (IQR 17-44). The median time from when the medical diagnosis and disposition decision were made to completion of EMR documentation was 9 minutes (IQR 5-16). The median time spent per patient from registration to final medical decision was 15 minutes (IQR 8-27). The overall potential EC burden was decreased significantly by 90.8%.

### Patient Outcomes

Outcomes were assessed as of April 13, 2020. Only 257 patients (9.2%) returned to the EC for an evaluation within 7 or more days, out of which 64 patients (24.9%) were admitted to the hospital. In total, 11 (17.2%) patients are still currently admitted, and 4 (6.2%) admitted patients have expired.

## Discussion

### Principal Findings

Based on our experience and previous published literature [7], we worked to address process limitations as they became apparent during curbside testing implementation. An important limitation that many facilities across the nation also faced was limited testing availability [8]. We developed educational materials, with information on when to get tested, that were available on our website. We also had phone and online screening questionnaires that were used to determine if a person needed to present for curbside testing. A facility should address this limitation by working to inform the population about limitations in resources and selective testing capabilities with focus on patients who are considered at increased risk of developing severe disease [5]. We anticipate that this problem will be mitigated as testing becomes more readily available. While we initially had long wait times for testing, we opened multiple triage and screening locations based on the surge of patients and also streamlined the documentation process, increased staffing, and processed in parallel instead of serially in order to address this issue. We also had to create solutions to caring for medically unstable patients. We recommend having a separate triaging location from the screening location in order to identify patients at high risk for severe disease and direct them in a timely manner to receive traditional EC care. In addition, we tested patients in early spring, which is often associated with cold temperatures in Michigan. A large outdoor space was required for this curbside model in order to mitigate the high risk of contagiousness. However, an area with warming potential needs to be chosen to ensure the protection of personnel from the outdoor conditions. This will become relevant if another wave of COVID-19 occurs this upcoming fall and winter. We did not record and quantify the number of patients that were triaged and sent straight to the EC to be evaluated; therefore, we cannot report on the actual number of patients who sought EC curbside testing in the first place.

### Conclusion

Curbside screening has been shown to be safe for COVID-19 patients. The process is also efficient, with a median of 15 minutes spent per patient from registration to final medical decision. Our findings support the incorporation of this model at other high-volume facilities during an infectious disease pandemic.

## Abbreviations

APP: advanced practice provider
CDC: Centers for Disease Control and Prevention
COVID-19: coronavirus disease
EC: emergency center
EMR: electronic medical record
PPE: personal protective equipment
RT-PCR: reverse transcriptase–polymerase chain reaction
SARS-CoV-2: severe acute respiratory syndrome coronavirus 2
WHO: World Health Organization

## References

[1] Rothan HA, Byrareddy SN (2020). The epidemiology and pathogenesis of coronavirus disease (COVID-19) outbreak. J Autoimmun.

[2] Cucinotta Domenico, Vanelli M (2020). WHO Declares COVID-19 a Pandemic. Acta Biomed.

[3] (2020). Coronavirus - Michigan Data. Michigan.gov.

[4] Meltzer MI, Cox NJ, Fukuda K (1999). The economic impact of pandemic influenza in the United States: priorities for intervention. Emerg Infect Dis.

[5] Emergency Medical Treatment & Labor Act (EMTALA). Centers for Medicare & Medicaid Services.

[6] COVID-19 Emergency Center Curbside Screening. Beaumont Health.

[7] Kwon KT, Ko J, Shin H, Sung M, Kim JY (2020). Drive-Through Screening Center for COVID-19: a Safe and Efficient Screening System against Massive Community Outbreak. J Korean Med Sci.

[8] (2020). Previous U.S. Viral Testing Data. Centers for Disease Control and Prevention.

